# Dual-band-enhanced Transmission through a Subwavelength Aperture by Coupled Metamaterial Resonators

**DOI:** 10.1038/srep08144

**Published:** 2015-01-30

**Authors:** Yunsheng Guo, Ji Zhou

**Affiliations:** 1 State Key Laboratory of New Ceramics and Fine Processing, School of Materials Science and Engineering, Tsinghua University, Beijing 100084, China; 2 Department of Physics, Inner Mongolia University of Science and Technology, Baotou 014010, China

## Abstract

In classical mechanics, it is well known that a system consisting of two identical pendulums connected by a spring will steadily oscillate with two modes: one at the fundamental frequency of a single pendulum and one in which the frequency increases with the stiffness of the spring. Inspired by this physical concept, we present an analogous approach that uses two metamaterial resonators to realize dual-band-enhanced transmission of microwaves through a subwavelength aperture. The metamaterial resonators are formed by the periodically varying and strongly localized fields that occur in the two metal split-ring resonators, which are placed gap-to-gap on either side of the aperture. The dual-band frequency separation is determined by the coupling strength between the two resonators. Measured transmission spectra, simulated field distributions, and theoretical analyses verify our approach.

Electromagnetic metamaterials are artificial structures designed to exhibit exotic properties that are unlike those of any conventional materials^1^,^2^,^3^,^4^. These materials have recently become of considerable interest because of their potential applications, such as their use in negative refractive index materials^2^,^3^, perfect lensing^4^, and cloaking^5^,^6^. Indeed, just as the usual characteristics of naturally occurring materials are derived from the properties of atoms or molecules^7^, the unusual characteristics of metamaterials also originate from the properties of meta-atoms^2^,^8^ or metamolecules^9^,^10^-the subwavelength resonance units, which are generally referred to as metamaterial resonators^11^,^12^,^13^. There are many types of resonators that adopt different resonance mechanisms, such as the *LC* resonance of metal split rings^14^,^15^,^16^, the plasmon resonance of metal thin rods^17^,^18^,^19^, the Mie resonance of dielectric ceramic particles^20^,^21^,^22^,^23^, and the ferromagnetic resonance of ferrites^10^,^18^,^19^, for engineering and assembling metamaterials. To date, most of the novel electromagnetic behaviors have been determined to be the collective response of abundant metamaterial resonators to external excitation fields. Owing to the strongly localized field within and around the subwavelength resonance unit (specifically, the metamaterial resonator strongly interacts with the incident wave when it reaches the resonant state), only one or two meta-atoms should have some unprecedented applications, unlike a real atom, for which the interaction of the field is very weak^24^.

Since extraordinary optical transmission through two-dimensional (2D) aperture arrays perforated in optically thick silver films was first discovered^25^, enhanced transmission at a certain wavelength has been investigated both theoretically and experimentally^26^,^27^. In addition to 2D aperture arrays, a single metallic subwavelength aperture can also achieve enhanced transmission^28^,^29^. It has been exposed that the resonance transmission of electromagnetic waves plays an important role in the emergence of enhanced transmission^30^. Now that various resonance units are available for metamaterial resonators, we are inspired to use their resonance mechanisms to increase the amount of light that passes through a single aperture. This question has been solved through coupled Mie resonances of two ceramic cube particles with high permittivity and with low loss^20^. In addition, multiband-enhanced transmission has various potential applications, such as in multi-wavelength sensors or filters. Therefore, considerable attention should be focused on multiband-enhanced transmission. Much of the work related to this topic has been conducted in the optical band through the use of multiple plasmon resonances in one metal structure^31^,^32^,^33^. However, at microwave frequencies, metals are perfect conductors, and thus, surface plasmons do not contribute to the enhancement process^16^. Therefore, exploring the multiband-enhanced transmission of microwaves through a subwavelength aperture is of great importance.

In this study, by using two metamaterial resonators placed symmetrically on either side of a subwavelength aperture, we experimentally realized the enhanced transmission of microwaves at two resonance frequencies. One frequency is lower than the fundamental frequency of an individual metamaterial resonator, and the other frequency is higher than the fundamental frequency. The two coupled metamaterial resonators work in-phase in the lower frequency mode, and they work out-of-phase in the higher frequency mode. We also present results from simulations and theoretical analyses, and these results are shown to be consistent with the experimental results.

## Results

**Figure 1:**
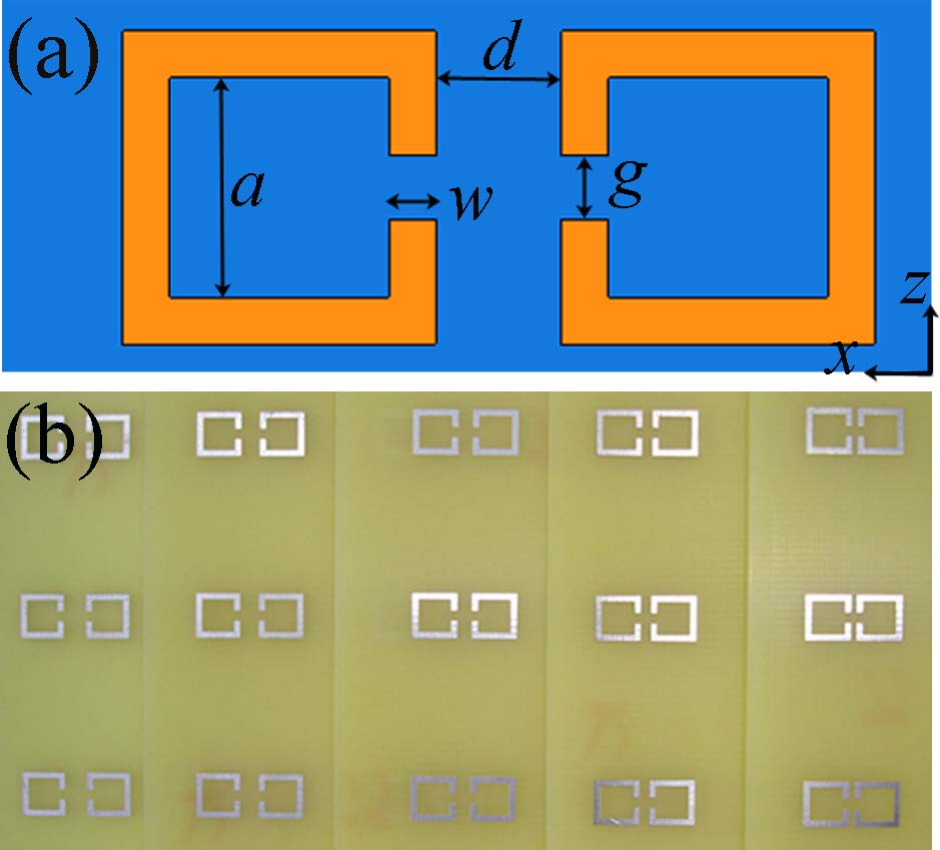
Sample characterization. (a) Schematic of a unit cell of the designed coupled SRR pair, in which yellow and blue represent the metal and substrate materials, respectively. (b) Photograph of one side of the fabricated coupled SRR array patterns with *d* *= * 2.5, 2.0, 1.5, 1.0 and 0.5 mm.

The metamaterial resonators used to realize dual-band-enhanced transmission are shown in Fig. 1. At first glance, these resonators appear to be metal split-ring resonators (SRRs), which are extensively used in left-handed materials^14^ or metamaterials^15^. The metal gap is equivalent to a capacitor because a large number of charges with equal quantity and opposite signs accumulate at both sides of the gap. The wire itself is a fraction of one winding of a magnetic coil. Under excitation by an external field, the SRR forms an *LC* resonance. However, when the two SRRs are arrayed gap-to-gap, as shown in Fig. 1 (a), energy can be transferred from one resonator to the other through the coupling electric and magnetic fields between the two resonators. Therefore, the proposed coupled metamaterial resonators are the time-varying fields that are strongly localized at the two SRRs. The geometrical parameters of the coupled square SRRs shown in Fig. 1(a) are *a* = 3.5 mm, *w* = 0.75 mm, *g* = 1 mm, and *d* = 2 mm. The dual-band frequency separation is determined by the coupling strength of the two metamaterial resonators, which is dependent on the space between the two SRRs; thus, *d* can be varied to obtain dual-band-enhanced transmission at different frequencies. For the experiment, a commercially available 1-mm-thick FR4 printed circuit board (with a dielectric constant of 4.4 and a loss tangent of 0.01) with a thin (18 μm) deposited copper plate layer was used to fabricate the SRRs. Fig. 1(b) presents a photograph of the fabricated coupled SRR array patterns, which include five different coupling spaces. Because only one coupled SRR pair unit is needed for investigating its performance on the transmission coefficients and the frequency separation of the dual-band-enhanced transmission through an aperture, the five coupled SRR array patterns were cut into unit cells with dimensions of 15 × 6 mm. The resonance frequency of a single SRR is required; thus, some single SRR units with dimensions of 6 × 6 mm were also prepared.

**Figure 2:**
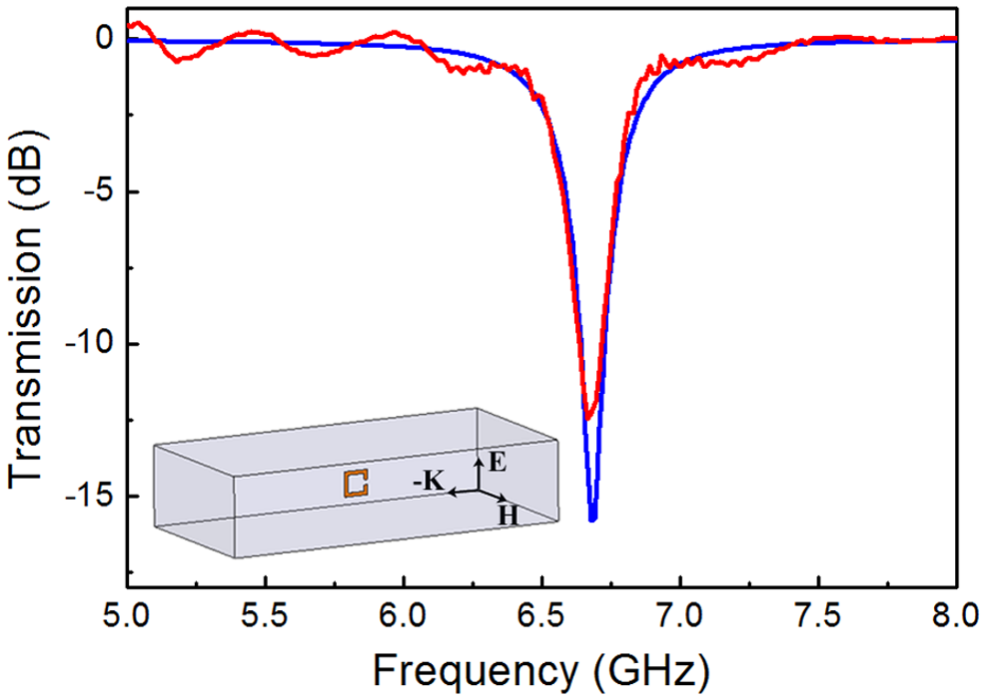
Microwave transmission spectrum of a single SRR unit. The red line represents the measured spectrum, and the blue line represents the simulated spectrum. The inset shows a schematic of a microwave measurement system that uses a rectangular waveguide.

**Figure 3:**
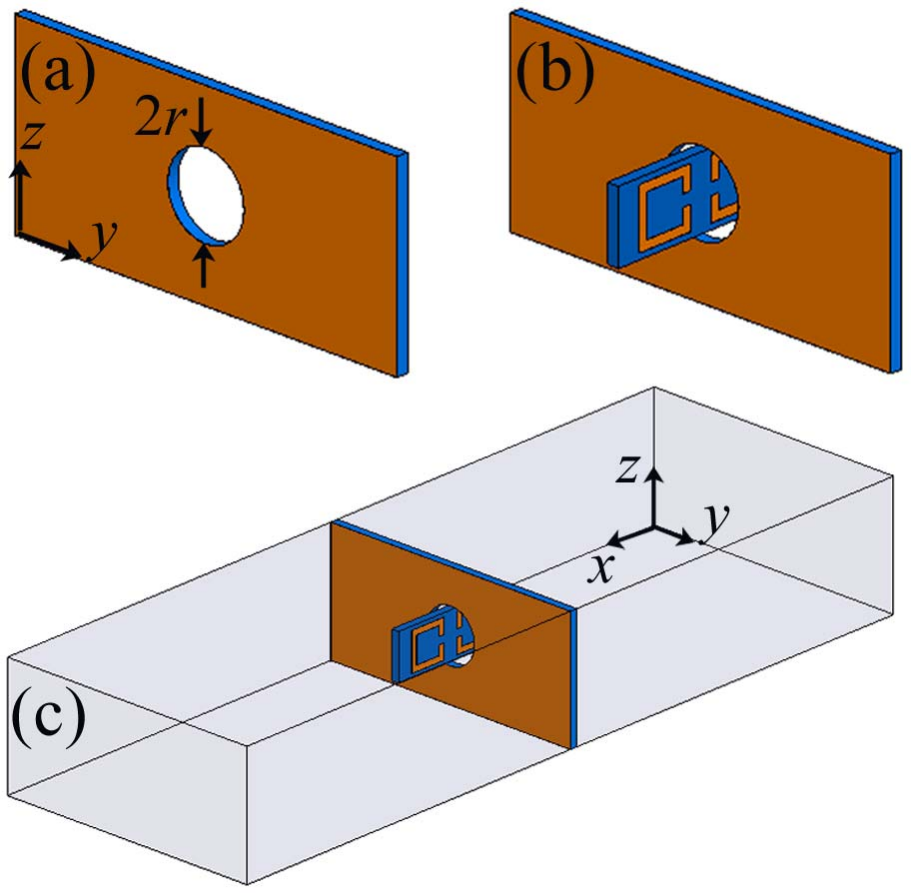
Schematics of the subwavelength aperture and experimental setup. (a) A subwavelength aperture with a radius of *r* = 3.5 mm at the center of a copper plate with dimensions of 70 × 50 mm. (b) A coupled SRR pair unit cell inserted in the subwavelength aperture. (c) Microwave measurement system for dual-band-enhanced transmission using two rectangular waveguides separated by the copper plate containing the subwavelength aperture.

The microwave transmission spectrum shown in Fig. 2 indicates that the resonance frequency of the single SRR unit is 6.7 GHz, and the measured and simulated results are in agreement. Here, the resonance frequency can be regarded as the fundamental frequency by analogy to a pendulum. Fig. 3 shows the schematics of the subwavelength aperture and of the microwave measurement system for dual-band-enhanced transmission. The metal aperture in Fig. 3(a) was also manufactured from the aforementioned printed circuit board. The radius of the aperture is 3.5 mm, and the resonance frequency of the single SRR is 6.7 GHz; thus, the aperture fully satisfies the subwavelength requirement (*r*/*λ* = 0.08). The two coupled metamaterial resonators were placed symmetrically on either side of the aperture, as shown in Fig. 3(b). The dual-band-enhanced transmission was measured using two rectangular waveguides, as schematically illustrated in Fig. 3(c).

**Figure 4:**
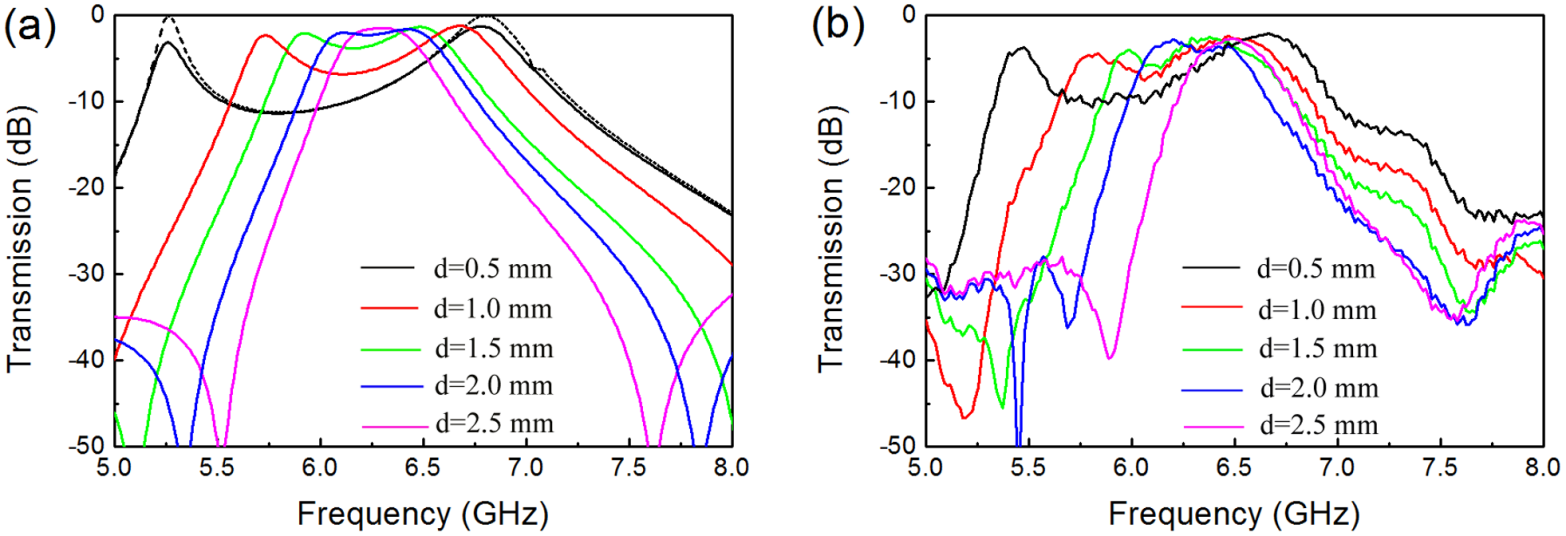
Dual-band-enhanced transmission spectra of microwaves through a subwavelength aperture. (a) Simulated transmission spectra with different *d*. The solid black line represents the results when all materials used are ideal. (b) Measured transmission spectra with different *d*.

Fig. 4 presents the microwave transmission spectra of the coupled metamaterial resonators placed on either side of the aperture. The transmission coefficients with the different coupling spacings are shown. We clearly observe two transmission peaks that arise at frequencies lower and higher than the fundamental frequency of the single SRR. Moreover, as the coupling space increases, the dual-band frequency separation becomes increasingly narrower until the two frequency bands begin to merge at the fundamental frequency. For example, in the simulation results shown in Fig. 4(a), for the spacing of *d* = 0.5 mm, the transmission coefficients reach up to −3.1 and −1.2 dB at 5.26 and 6.78 GHz, respectively. At the spacing of *d* = 1.0 mm, they are −2.3 dB at 5.73 GHz and −1.2 dB at 6.68 GHz. When the spacing is *d* = 2.5 mm, the two frequency bands begin to merge at 6.43 GHz, and the transmission coefficient is −1.4 dB. Note that the transverse dimension of the plane wave front (or the size of the waveguide) has a certain impact on the transmission coefficient and resonance frequency. When a subwavelength metal aperture is placed in the middle of the two metamaterial resonators, the fundamental frequency of the resonator has changed into 6.43 GHz from 6.7 GHz. The measured results shown in Fig. 4(b) and the simulation results shown in Fig. 4(b) are consistent overall, and the differences may be a result of fabrication errors and inaccurate placement of the coupled SRR pair. The transmission coefficient at *d* = 0.5 mm is indicated by the solid black line in Fig. 4(a) for the case when the printed circuit board is assumed to be lossless. We observe that the transmission coefficient is almost 0 dB (actually, −0.1 dB) at the two frequency bands, indicating that no reflection is occurring, despite the presence of the metal plate with the subwavelength aperture. Therefore, dual-band-enhanced transmission through a subwavelength aperture based on the coupled metamaterial resonators is obtained.

**Figure 5:**
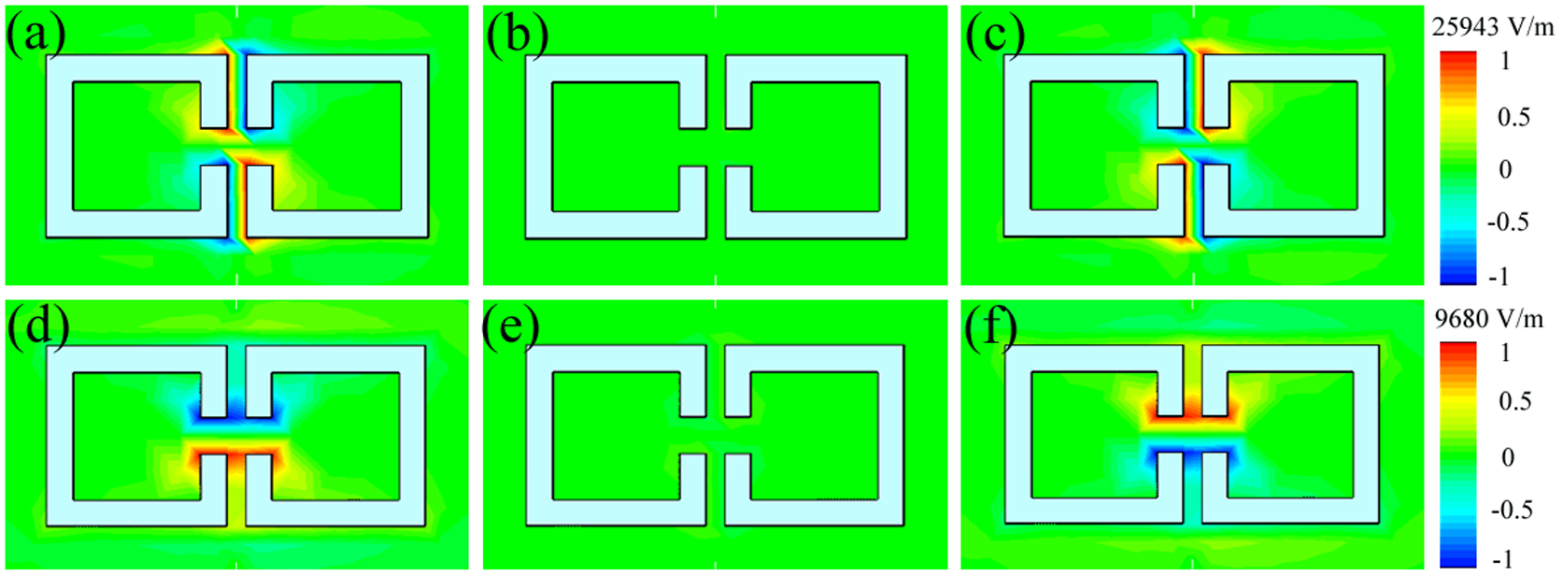
Simulated electric field intensity distributions of the coupled metamaterial resonators at the two resonant modes. Distribution results at phase angles of (a) *θ* = 0°, (b) *θ* = 90°, and (c) *θ* = 180° at the lower frequency of 5.26 GHz and those at phase angles of (d) *θ* = 0°, (e) *θ* = 90°, and (f) *θ* = 180° at the higher frequency of 6.78 GHz.

To further explore the mechanism for the dual-band-enhanced transmission of microwaves through a subwavelength aperture by the two coupled metamaterial resonators, we calculated the time-varying electric field intensities of the two resonators at lower and higher frequencies. The animated simulation results (data not shown) indicated that, in the lower frequency mode, the currents in both SRRs are in-phase; however, in the higher frequency mode, the currents in both SRRs are antiphase (180° out-of-phase). Fig. 5 shows the static electric field distributions calculated at phase angles of *θ* = 0°, 90°, and 180° for the incident wave when the coupling space is *d* = 0.5 mm. We clearly see that the electric field intensities in the two metamaterial resonators at 5.26 GHz simultaneously achieve maxima at *θ* = 0° in Fig. 5 (a), zeros at *θ* = 90° in Fig. 5(b), and reversed maxima at *θ* = 180° in Fig. 5(c). If we form a loop along the direction of the electric field in either SRR, we find that the directions of the two loops are identical. Therefore, the two metamaterial resonators are in-phase. However, if we use the same method to compare the directions of the currents in both SRRs in Figs. 5(d), 5(e), and 5(f), we observe that the two metamaterial resonators are out-of-phase. In short, the two metamaterial resonators are in-phase in the lower frequency mode and out-of-phase in the higher frequency mode.

## Discussion

In this work, meta-atoms (metamaterial resonators) are formed by the periodically varying and strongly localized fields that occur within and around SRRs. By placing two SRRs gap-to-gap on either side of an aperture, dual-band (the lower frequency is in in-phase mode, and the higher frequency is in antiphase mode)-enhanced transmission is realized. In addition, the fundamental frequency of an individual SRR is dependent on the scale of the plane wave front, the SRR and its gap, and the frequency separation of the two coupled modes is determined by the coupling space. Therefore, by adjusting the size of the waveguide, the dimensions of the SRRs, the gaps of the SRRs, and the coupling space, dual-band-enhanced transmission at the desired microwave frequencies can easily be attained. This approach will help enable multiband-enhanced transmission of microwaves through a subwavelength aperture.

## Methods

### Theoretical description

**Figure 6:**
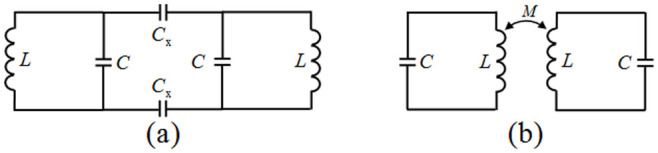
Equivalent circuits of the coupled metamaterial resonators. (a) Equivalent circuit coupled by two capacitors *C*_x_. (b) Equivalent circuit coupled by a mutual inductance *M*.

The resonance frequencies of two pendulums connected by a spring can be analyzed using complex dynamics theory. The results show that the separation between the lower and higher frequencies increases with increasing coupling strength^34^. Analogously, the presented dual-band-enhanced transmission of microwaves through a subwavelength aperture can be described using microwave circuit theory. Fig. 6 presents schematic illustrations of the two metamaterial resonators coupled by the electric and magnetic fields. Fig. 6(a) is the equivalent electrical circuit coupled by the electric fields, which consists of two *LC* circuits plus two coupling capacitors *C*_x_ between the two SRRs. The ohmic losses are ignored because the copper is close to a perfect conductor at the microwave band. By using the Pi-Tee conversion, we can obtain the resonance frequencies in Fig. 6(a) as^34^ *ω*_1_^2^ = 1/*L*(*C* + *C*_x_) and *ω*_2_^2^ = 1/*LC*, which indicate that the lower resonance frequency decreases with an increase in the capacitance of the coupling capacitors *C*_x_, and that the higher resonance frequency is independent of the coupling strength. Fig. 6(b) is the equivalent electrical circuit coupled by the magnetic fields, which consists of two *LC* circuits plus a mutual inductance *M* existing between the two inductors *L*. The two resonance frequencies are^35^,^36^,^37^ *ω*_1_^2^ = 1/(*LC*(1 + *k*)) and *ω*_2_^2^ = 1/(*LC*(1 − *k*)), where *k*^2^ = *M*^2^/*L*^2^ is the coupling coefficient. This shows that the lower resonance frequency decreases with an increase in the inductance of the coupling inductor, and the higher resonance frequency increases with an increase in the inductance of the coupling inductor.

**Figure 7:**
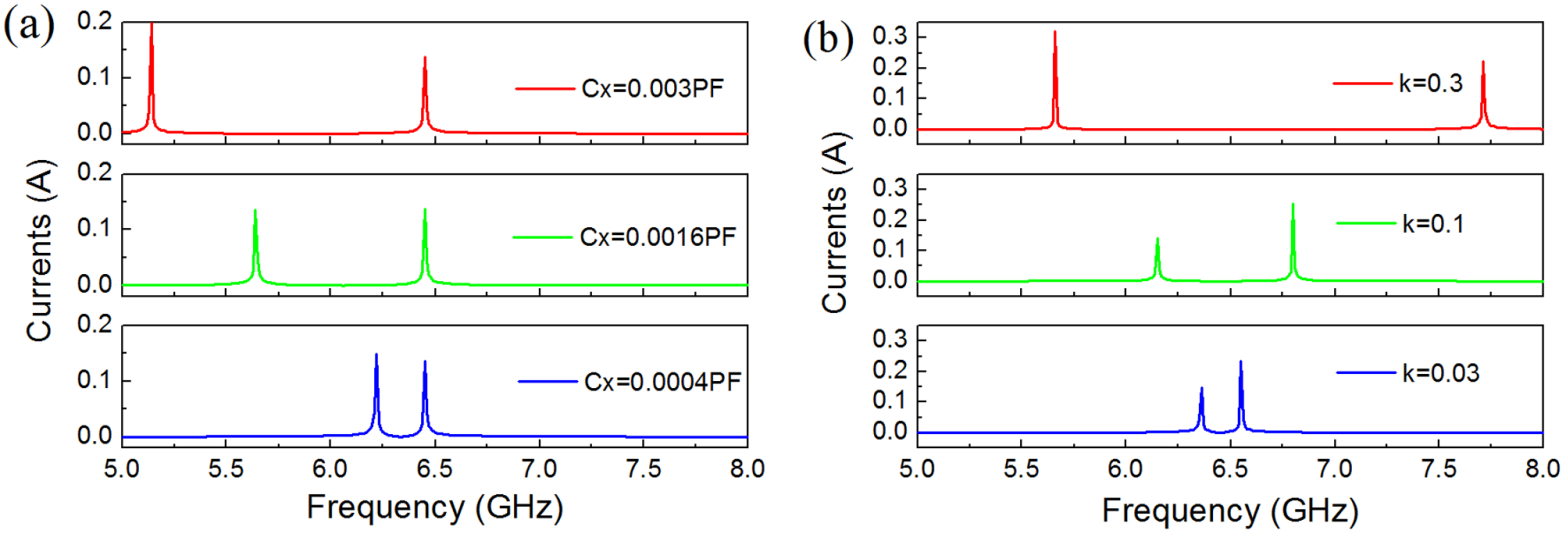
Currents through the equivalent electrical circuits with different coupling coefficients. (a) Circuits coupled by different capacitors *C*_x_. (b) Circuits coupled by different inductors *M*.

In order to demonstrate that the resonance frequencies of the two coupled metamaterial resonators change as a function of the coupling, we numerically calculated the resonance frequencies at different coupling coefficients. Fig. 7 shows the currents through the equivalent electrical circuits coupled by different capacitors *C*_x_ and inductors *M*. Here the supply voltage is assumed to be 1 V, and the equivalent capacitor *C* and inductor *L* are 5.2 × 10^−3^ PF and 117 nH obtained from formulas of the lumped elements for microwave circuits^38^. We can see that, no matter what kind of coupling, the frequency separation of the two resonant currents increases with increasing coupling strength. However, when the two metamaterial resonators are coupled by the electric fields, the higher resonance current is irrelevant to the coupling strength. From the simulated and measured results shown in Fig. 4, we know that the frequency shift occurs simultaneously on the lower and higher resonance modes. Therefore, we can conclude that dual-band-enhanced transmission should be attributed to the hybridization effect of the electric and magnetic couplings. The frequency shift of the higher resonance mode is smaller than that of the lower resonance mode, thus we think that the lower resonant mode is mainly dependent on the electric coupling and the higher one is mainly dependent on the magnetic coupling. In addition, the transverse size of the waveguide has a certain impact on the fundamental frequency of the single metamaterial resonator and then on the dual resonance frequencies of the coupled metamaterial resonators. One must take the effect of waveguide into account before realizing dual-band-enhanced transmission of microwaves through a subwavelength aperture.

**Figure 8:**
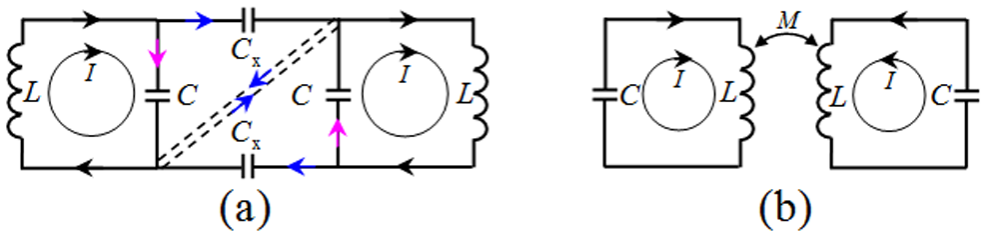
Currents through the circuits at two resonance frequencies. (a) At lower frequency *ω*_1_^2^ = 1/*L*(*C* + *C*_x_), and (b) at higher frequency *ω*_2_^2^ = 1/(*LC*(1 − *k*)). Arrows with different colors represent currents with different values.

Finally, based on the expressions of the resonance frequencies of the circuits shown in Fig. 6, we highlighted the currents through the circuits at lower and higher frequencies in Fig. 8. They clearly show that the two metamaterial resonators are in-phase at the lower frequency in Fig. 8(a) and out-of-phase at the higher frequency in Fig. 8(b). The theoretical analysis is consistent with the simulation result.

### Sample preparation and measurement

SRR array patterns with the five different coupling distances were etched on FR4 printed circuit boards using conventional photolithography techniques. The single SRR samples with dimensions of 6 × 6 mm, the coupled SRR pair samples with dimensions of 15 × 6 mm, and the metallic plate with dimensions of 70 × 50 mm were all cut using a precision automatic dicing saw (HP-603, China). The subwavelength aperture with a radius of 3.5 mm at the center of the metallic plate was created by mechanical etching. The transmission spectra of the single SRR and of the dual-band-enhanced transmission through a subwavelength aperture based on the coupled metamaterial resonator pair were measured using two linked WR-137 rectangular waveguides with sectional dimensions of 34.85 × 15.80 mm. The other ends of the two waveguides were connected to the input and output of a vector network analyzer (N5230C, Agilent Technologies, USA). The simulated microwave transmission spectra and electric field distributions were obtained using the Microwave Studio software package (CST Studio Suite 2011, Germany). The microwave circuit simulations were performed using Advanced Design System 2009 (Agilent Technologies, USA). The material and structural parameters for the simulation modes were fully consistent with the experimental values.

## Author Contributions

Y.S.G. and J.Z. conceived the idea and designed the experiments. Y.S.G. performed the experiments, developed the post-processing treatments of the experimental data, carried out the numerical calculations and created the figures. Y.S.G. and J.Z. wrote the paper. Both authors contributed to scientific discussions and critical revisions of the article.
